# Bridging stakeholder needs and technological capabilities to identify humanoid robot applications in rehabilitation: a case study in an ALS multidisciplinary care unit

**DOI:** 10.3389/frobt.2026.1944029

**Published:** 2026-09-21

**Authors:** Rachele Piras, Samuele Bordini, Riccardo K. Simi, Ilaria Consolo, Valentina Esposito, Claudia Cavaggion, Maddalena Feder, Maria Fossati, Giorgio Grioli, Antonio Bicchi, Christian Lunetta, Manuel G. Catalano

**Affiliations:** 1 Istituti Clinici Scientifici Maugeri IRCCS, Department of Neurorehabilitation, Milano Institute, Milan, Italy; 2 Centro Di Ricerca E. Piaggio E Dipartimento Di Ingegneria Dell’Informazione, Università Di Pisa, Pisa, Italy; 3 Soft Robotics for Human Cooperation and Rehabilitation, Istituto Italiano di Tecnologia, Genova, Italy; 4 Fraunhofer Italia - IEC Bolzano, Bolzano, Italy; 5 Physical AI Technologies for Human-Robot Coevolution, Istituto Italiano di Tecnologia, Genova, Italy

**Keywords:** amyotrophic lateral sclerosis, clinical translation, humanoid robots, rehabilitation and assistive robotics, stakeholder-driven design

## Abstract

The integration of assistive service robots in healthcare has gained increasing attention for its potential to support clinical workflow and patient wellbeing. However, structured methodological approaches to identify clinically relevant applications for versatile robotic platforms before clinical implementation are still lacking. Moreover, the deployment of robots in these settings is often restricted to hyper-specialized tasks. To address these challenges, this study describes a stakeholder-driven process adopting a multidisciplinary co-design approach to adapt the Alter-Ego semi-anthropomorphic robot to the needs of a multidisciplinary neurorehabilitation unit for patients with Amyotrophic Lateral Sclerosis. We combined brainstorming, focus groups with healthcare professionals, patients and caregivers, and contextual observations to identify the real unmet needs and potential applications. The engineering team assessed the technological requirements and implementation readiness of each application. The clinical and engineering teams jointly reviewed the proposal, considering clinical, ethical, and infrastructural implications. This consensus-based process resulted in 12 use cases selected for the clinical protocol corresponding to 40 planned experimental sessions including autonomous or semi-autonomous tasks, as well as teleoperated activities for assistive and rehabilitation purposes. Furthermore, an exploratory pilot evaluated a subset of selected use cases, providing feedback to refine the platform and experimental protocol. Standardized assessments provided descriptive information on perceived workload, usability, and satisfaction. Participants generally reported a positive experience and manageable workload, while task-specific feedback identified aspects requiring further refinements. Overall, this experience offers preliminary insights into how clinical needs and technological capabilities can be jointly explored, providing a basis for subsequent clinical experimentation.

## 1 Introduction

Assistive technologies and service robots are increasingly being introduced into healthcare to support clinical activities, improve operational efficiency, and enhance patient care (Reich and Eyssel, 2013) in both domestic and clinical settings. These solutions have been recognized as promising tools to alleviate healthcare’s workload and support patient wellbeing (AlQudah et al., 2021; Lee et al., 2025). Despite these rapid technological advances, however, the translation of robotic systems from research laboratories into routine clinical practice remains limited (Rasmussen et al., 2024).

Among these technologies, robotic avatars have attracted increasing interest because they enable healthcare professionals to interact remotely with patients and clinical environments (Siciliano and Khatib, 2020), a capability that proved particularly valuable during the COVID-19 pandemic through telepresence applications (Fossati et al., 2020). Recent developments in artificial intelligence have further expanded robot capabilities, enabling autonomous navigation, language processing, and more natural human–robot interaction to support healthcare professionals in clinical practice (Garcia-Gonzalez et al., 2022; Perrin et al., 2010).

Humanoid and socially assistive robots, including platforms such as Pepper and NAO, have also been investigated in healthcare, rehabilitation, and elderly-care settings, providing relevant evidence regarding their usability, acceptability, and integration into care environments (Papadopoulos et al., 2022; Pino et al., 2020).

However, most existing robotic platforms in healthcare (Ohneberg et al., 2023) are still designed to perform single, predefined tasks, such as chatbots or telepresence, limiting their adaptability to the heterogeneous and dynamic needs of clinical practice. In addition, conventional rigid actuation restricts safe physical interaction with people and complex healthcare environments (Zahedi et al., 2022; Ilias et al., 2014; González-González et al., 2021).

Soft robotics, inspired by human biomechanics, is helping overcome these limitations by enabling safer and more adaptive interaction, thereby supporting the development of robotic platforms capable of performing a broader range of healthcare activities (Della Santina et al., 2021).

The Alter-Ego robot (Lentini et al., 2019), developed by the Department of Information Engineering at the University of Pisa in collaboration with the Italian Institute of Technology (IIT), exemplifies this new generation of versatile robots. Designed to operate in teleoperated, semi-autonomous, or fully autonomous modes, depending on the specific application, Alter-Ego combines a mobile base with compliant anthropomorphic arms (Catalano et al., 2011) and adaptive hands (Catalano et al., 2014), enabling the safe manipulation of objects of various shapes and sizes. These features, together with its human-like embodiment, make Alter-Ego particularly suitable for healthcare environments, where it may support healthcare professionals in activities such as delivering medications, food, and other items while enabling intuitive interactions with patients. Owing to its versatility, Alter-Ego represents a promising platform for a broad range of assistive applications in both hospital and home settings. Amyotrophic Lateral Sclerosis (ALS) represents an appropriate clinical model for evaluating these new versatile technologies. Among neurological disorders, ALS can be considered a paradigm of clinical and organizational complexity, as its progressive nature leads to severe motor impairment, respiratory failure, and bulbar dysfunction affecting speech and swallowing, requiring continuous multidisciplinary care, rehabilitation, monitoring, and assistance across both hospital and home settings. This combination of diverse and evolving clinical needs places a substantial burden on patients, caregivers, and healthcare professionals. Consequently, identifying robotic applications capable of supporting care in ALS provides a particularly demanding testbed for versatile assistive technologies, with solutions that may, following appropriate adaptation, be transferable to a broad range of other neurological and chronic conditions. In this context, Alter-Ego offers a promising solution to support healthcare professionals in the delivery of multidisciplinary care across hospital and home settings. The primary objective of this study is to identify clinically relevant and technically feasible assistive applications of the Alter-Ego robot to support healthcare professionals within a multidisciplinary ALS neurorehabilitation unit, considering the practical requirements for safe, usable, and operationally feasible deployment in clinical practice.

Achieving this objective requires a systematic methodology capable of translating clinical needs into robotic applications. However, while previous studies have primarily assessed user acceptance via surveys or brief interviews (Mlakar et al., 2025; Cresswell et al., 2018) or explored experience-based participatory robotics for single, predefined tasks (Gasteiger et al., 2022; Gebellí and Ros, 2025), collaborative clinician–roboticist co-design remains uncommon, and structured methodological approaches to identify, prioritize, and refine multiple clinically relevant applications of a versatile robotic platforms across a disease-specific multidisciplinary care pathway before clinical implementation are still lacking.

To address this gap, our methodological approach was inspired by the principles of participatory design and co-design proposed by Sanders and Stappers (Sanders and Stappers, 2008), where knowledge emerges through collaboration among stakeholders with complementary expertise. In this study, healthcare professionals represent the primary end-users of the robotic platform, whereas patients are considered key stakeholders and beneficiaries of the services delivered through it. Building upon previous preclinical studies on the assistive use of Alter-Ego in hospital and domestic environments (Piras et al., 2024), this work integrates multidisciplinary clinical expertise, stakeholder engagement, contextual observations, and technological feasibility assessment to identify meaningful use cases. An exploratory pilot phase was incorporated to assess their feasibility and optimize both the robotic platform and the experimental protocol before subsequent clinical trials.

## 2 Methods

This study employs a multidisciplinary approach to assess the integration of the robotic platform Alter-Ego, into the rehabilitative and assistive care context to support healthcare professionals.

The proposed methodological framework goes beyond a stakeholder-driven process, adopting a multidisciplinary co-design approach based on shared decision-making throughout all stages of development, through the continuous interaction between the engineering team and the multidisciplinary healthcare professionals’ team. The approach brought together engineering expertise, clinical knowledge, and the practical experience of the healthcare professionals whose workflows would be affected by the implementation of the robotic platform. In this study, healthcare professionals were considered the primary end-users, whereas patients and caregivers, who ultimately benefit from the services provided through the platform, were involved as key stakeholders to ensure, through their perspective, the alignment between professional requirements and patient expectations. Although both groups actively participated in the process, they fulfilled complementary roles reflecting the objectives of the project. The study originated from brainstorming sessions between the ALS expert team of ICS Maugeri IRCCS Milan Institute and the engineering team exploring the potential role of Alter-Ego to support healthcare professionals in the care of ALS patients in both hospital and home settings.

The methodological approach followed a sequential process described below and shown in Figure 1.

**FIGURE 1:**
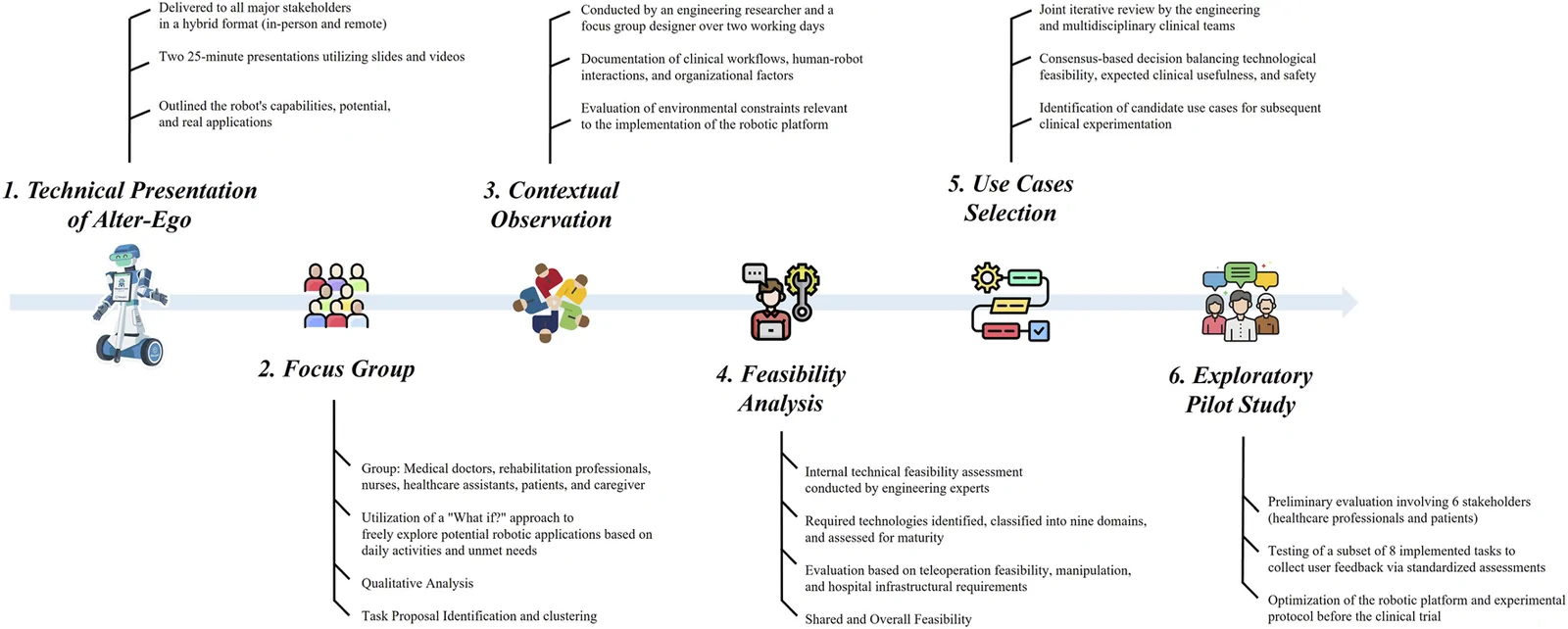
Schematic representation of the stakeholder-driven co-design methodological framework. The process consists of six phases: (1) A technical presentation of the Alter-Ego robotic platform to outline its capabilities and potential; (2) Multidisciplinary focus groups involving stakeholders to identify real-world needs and proposed tasks; (3) Contextual observations within the rehabilitative setting to analyze interactions between the robot, the environment, and the end-users; (4) an internal feasibility analysis conducted by engineering experts to assess the technical viability of the proposed tasks; (5) a consensus-based use cases selection jointly performed by engineering and clinical teams to identify priority applications for subsequent experimentation; and (6) an exploratory pilot study to collect preliminary user feedback, assess technological maturity, and optimize procedures before the clinical trial.

### 2.1 Technical presentation of Alter-Ego

To maximize stakeholder participation, two identical 25-min technical presentations were delivered in a hybrid format (in-person and remote), including slides and videos that outlined the robot’s capabilities, potential, and real applications. A clear and accessible language was used to ensure a common understanding. Questions and discussions at the end of each session encouraged active engagement.

### 2.2 Focus groups and task categorization

Focus groups were conducted with the main stakeholder groups: medical doctors (3 neurologists and 2 physiatrists (n = 5), rehabilitation professionals (11 physiotherapists, 3 occupational therapists, 1 respiratory therapist, 3 speech therapists: n = 18), nurses and healthcare assistants (n = 10: 5 + 5). Each professional category participated in a dedicated session to capture profession-specific perspectives. 5 patients in different stages of disease were interviewed one-to-one to accommodate differences in disease severity and communication abilities; one individual interview was conducted with one caregiver. Each session lasted approximately 60 min.

All focus groups were conducted using a user-centred approach and moderated by an experienced facilitator who promoted an open, non-judgmental discussion, ensuring balanced participation among all participants. Following a brief review of the robot’s capabilities, discussions were conducted using a *What if?* approach, encouraging participants to freely explore potential robotic applications based on their daily activities, unmet needs, and care experiences.

All sessions were audio-recorded, transcribed verbatim, and reviewed by an ALS clinician and the designer who facilitated the focus groups. An inductive qualitative content analysis was conducted to systematically translate stakeholder contributions into candidate robotic applications.

First, all proposed activities were extracted from the transcripts and listed individually, regardless of how many stakeholder groups suggested the same activity. The proposals reflected concrete examples of potential robotic support in routine clinical practice, such as delivering personal items, assisting with remote rehabilitation, or monitoring symptoms.

The extracted activities were then examined to identify thematic areas representing conceptually related healthcare needs. Through an iterative consensus process, functional clusters emerged inductively from the data and were used to organize the proposals. Duplicate activities proposed by different stakeholder groups were merged, while conceptually similar activities were consolidated into broader application proposals. For example, delivering a bottle of water and delivering a television remote control were grouped into a single proposal concerning the delivery of small personal objects, whereas remote physiotherapy and remote speech therapy sessions were integrated into a single telerehabilitation proposal.

This process generated the application proposals, collected in columns A and B of Figure 2.

**FIGURE 2:**
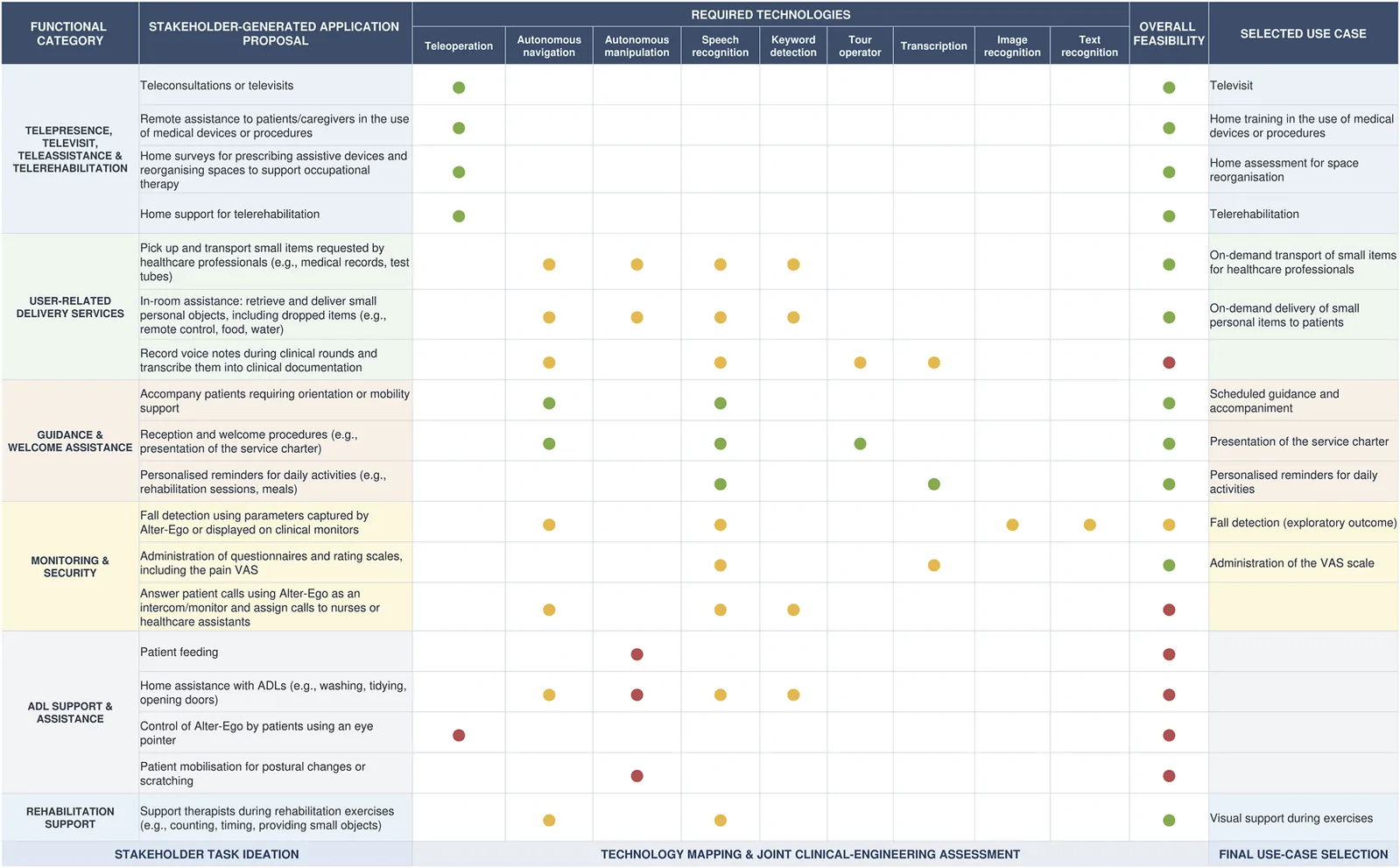
Task ideation, technology mapping, and feasibility assessment matrix. The evaluation process is structured into three main phases (bottom bar): (1) Stakeholder Task Ideation, where the 81 stakeholder suggestions were categorized into six Functional Categories and consolidated into 18 Stakeholder-Generated Application Proposals; (2) Technology Mapping and Joint Clinical-Engineering Assessment, which reports the Required Technologies for each proposed application, their current level of availability, and the Overall Feasibility; and (3) Final Use-Case Selection, establishing overall task viability and final effective use cases. In the technology columns, circles indicate whether each required technology was available (green), under development but implementable within the project timeframe (orange), or lacking (red). In the feasibility column, green indicates proposals considered feasible and appropriate for clinical experimentation; orange indicates the proposal selected as an exploratory outcome; red indicates proposals not retained following the multidisciplinary assessment. The final column (Selected Use Case) reports the 12 use cases selected for the experimental protocol. Further details are provided in the Supplementary Material.

### 2.3 Contextual observations

Contextual observations were conducted by an engineering researcher and the focus group facilitator over two non-consecutive working days, covering both morning and afternoon shifts in the ALS neurorehabilitation unit. Clinical workflows, interactions among healthcare professionals, patients and caregivers, environmental constraints, and organizational factors relevant to the implementation of the Alter-Ego platform were documented through field notes. The observations were useful for the technical feasibility analysis and were subsequently discussed within the multidisciplinary research team and integrated with the focus group findings to support the identification and prioritization of candidate robotic applications.

### 2.4 Feasibility analysis

An internal technical feasibility assessment was conducted by engineering experts to evaluate whether the proposed tasks could be executed with the available capabilities of the Alter-Ego platform or technologies implementable within a short timeframe.

For each candidate application, the technologies required for implementation were identified and classified into nine domains (Figure 2, column C). Their maturity was then assessed according to the current state of development and categorized as: (green) readily available, (orange) under development but ready in a reasonable timeframe, or (red) requiring further technological advancement, not compatible with the required timeframe.

Each candidate’s application was then evaluated according to these predefined criteria.

Teleoperation’s feasibility is based on the availability of dedicated, easy-to-use hardware for non-expert users, including an intuitive, practical, and wearable control interface, enabling operation of a high-degree-of-freedom robotic system. Autonomous tasks were evaluated according to the perception and understanding of hospital and home environments, which differ significantly from controlled laboratory settings, and considering the robot’s physical dimensions and intrinsic capabilities.

Manipulation tasks were assessed considering the robot’s workspace, reachability, payload, and object-grasping capabilities. Furthermore, it is fundamental to address the dimensions of the objects that the robot can grasp based on its hand geometry.

Infrastructural requirements for the deployment of the robotic platform in the hospital environment were also evaluated: compliance with hospital safety regulations, the availability of secure network connectivity, and the communication infrastructure required to support teleoperation and internet-based functionalities.

This technological readiness matrix provided the basis for the subsequent joint feasibility assessment. The applications were not assigned numerical scores or formally ranked. Each proposal was discussed iteratively by the engineering and clinical teams against the predefined domains of technical feasibility, clinical relevance, patient safety, ethical acceptability, regulatory compatibility, infrastructural requirements, and integration into clinical workflow. Final inclusion required shared agreement between the clinical and engineering teams. Final task prioritization resulted from this consensus-based decision-making process, balancing technological feasibility with clinical usefulness, patient safety, professional responsibilities, regulatory constraints, and implementation within routine clinical workflows. Applications were progressively refined and prioritized through consensus until the final set of candidate use cases was identified.

Proposals selected by consensus were presented to the patients and caregivers in one-to-one sessions for feedback regarding their perceived usefulness, relevance, and acceptability. This final consultation ensured that the selected applications remained aligned with patient priorities.

The selected use cases were subsequently translated into the experimental protocol. Because several use cases were evaluated by different professional groups or repeated under different clinical conditions (e.g., telemedicine delivered by different specialists or repeated pain assessment during routine care), the number of experimental sessions exceeded the number of distinct use cases.

### 2.5 Exploratory pilot phase

Following the identification of priority use cases and implementation of corresponding functionalities on Alter-Ego, a preliminary evaluation assessed technological maturity, optimized the operative protocol procedures, and collected stakeholders’ feedback before the clinical trial. Although the platform’s core hardware remains unchanged, a torso-mounted interface and custom software modules were implemented to support the exploratory clinical applications. Foundational hardware and software developments of the platform remain accessible through the open-source Natural Machine Motion Initiative^1^ (NMMI) (Della Santina et al., 2017). This phase involved 6 stakeholders: one neurologist, one physiotherapist, one speech therapist, and three patients.

Healthcare professionals were recruited on a voluntary basis from the multidisciplinary ALS Neurorehabilitation Unit. Patients were consecutively recruited among those admitted to the same unit during the study period following referral by their treating neurologists. Eligible patients were adults (18–80 years) with a diagnosis of ALS according to the revised El Escorial criteria, able to understand the study procedures and provide informed consent. Patients with moderate-to-severe cognitive impairment, severe psychiatric disorders, or conditions, according to medical judgement, preventing safe participation in the experimental sessions were excluded. Participation was voluntary for all participants, and written informed consent was obtained before enrolment.

A subset of 8 implemented tasks was evaluated, including: televisit, telerehabilitation with both the physiotherapist and speech therapist, small object delivery to patients and staff, welcome and guidance activities (service charter presentation and personalised reminders), and patient monitoring through the Visual Analogue Scale (VAS).

The demographic, professional, and clinical characteristics of the study participants, as well as the experimental tasks assigned to each participant, are presented in Table 1. Functional status was described using the Amyotrophic Lateral Sclerosis Functional Rating Scale–Revised (ALSFRS-R) (Cedarbaum et al., 1999), which provides a standardized measure of disability across bulbar, motor, and respiratory domains and enables characterization of the functional severity of the enrolled patients. The IT Familiarity Questionnaire (ITFQ) developed by Geyer, M. J (Faett et al., 2013). was used descriptively to characterize baseline participants’ familiarity with technologies. Before the experimental testing, healthcare professionals received a standardized 15-min training session on the use of the robotic platform. Both qualitative and quantitative feedback were collected. After each, task participants were asked to report their perceptions of the interaction with the robot, while usability, satisfaction, and perceived workload were evaluated using the System Usability Scale (SUS) (Brooke, 1996), the Questionnaire for User Interface Satisfaction (QUIS) (Chin et al., 1998), and the NASA Task Load Index (NASA-TLX) (Hart and Staveland, 1988), in its Raw version (Georgsson, 2020). Patients did not complete the SUS scale, as they did not directly interact with the teleoperator interface of the robotic platform.

**TABLE 1:** Participant characteristics, technological familiarity, and experimental tasks.

A. Demographic and clinical characteristics and experimental tasks								
Participant/role	Age	Sex	ALSFRS-R	Communication	Respiratory support	Mobility	Previous robotic experience	Task(s) performed
Patient 001	62	M	27	Mild dysarthria	None	Prevalent wheelchair user	No	Telerehabilitation with physiotherapist; televisit
Patient 002	59	F	28	Normal speech	None	Wheelchair user	No	Small-object delivery to patient; personalized reminders
Patient 003	26	F	24	Dysphonia	None	Ambulatory with aid/wheelchair user	No	Telerehabilitation with speech therapist; VAS monitoring; service charter presentation
Medical doctor	44	F	–	–	–	–	Yes	Televisit
Physiotherapist	34	F	–	–	–	–	Yes	Telerehabilitation; small-object delivery to staff; VAS monitoring; service charter presentation; personalised reminders
Speech therapist	27	F	–	–	–	–	Yes	Telerehabilitation

B. Information technology familiarity questionnaire									
Participant/role	Q1	Q2	Q3	Q4	Q5	Q6	Q7	Q8	Mean item score
Patient 001	3	3	2	2	1	2	3	2	2.25
Patient 002	1	1	1	2	3	1	1	3	1.63
Patient 003	1	1	1	1	1	1	1	1	1.00
Medical doctor	1	1	1	1	1	1	1	1	1.00
Physiotherapist	1	1	1	1	1	1	1	1	1.00
Speech therapist	1	1	1	1	1	1	1	1	1.00

Demographic and clinical characteristics, including Amyotrophic Lateral Sclerosis Functional Rating Scale–Revised (ALSFRS-R) scores, are reported for patients. Demographic and occupational characteristics were reported for healthcare professionals. Familiarity with information technology was assessed for all participants using the ITFQ. IT Familiarity Questionnaire items: Q1, send and receive emails; Q2, obtain information on a range of topics; Q3, download applications; Q4, shop, manage a calendar, or arrange travel; Q5, online banking and bill payment; Q6, social networking; Q7, watch videos, listen to podcasts or music, or share images; Q8, use other electronic technologies such as e-books or tablets. Response options were coded as follows: 1 = daily use, 2 = occasional use, and 3 = never used; therefore, lower mean scores indicate greater familiarity with information technology. Each participant could perform one or more tasks.

We utilized QUIS version 7.0, employing a 9-point semantic differential scale (1-9, with higher scores reflecting greater satisfaction). The evaluation includes five core dimensions: Screen, Terminology and System Info, Learning, System Capabilities, and General Impression. Scores for each dimension were calculated as the average of completed items individually for each subject. Questionnaires included a “not applicable” response option for items not relevant to the participant’s role, tested use case, or specific interaction with the platform that were treated as missing data and strictly excluded from the calculations.

Given the exploratory nature of this pilot phase and the limited sample size, questionnaire data were not intended for statistical inference or for the calculation of overall usability indices. Rather, individual responses were analyzed to identify task-specific strengths and critical issues, gather stakeholders’ feedback, and support the refinement of the Alter-Ego platform, the assessment protocol, and the Standard Operating Procedures (SOPs) for the subsequent clinical trial.

## 3 Results

### 3.1 Focus group

The focus groups generated 81 stakeholder suggestions for potential applications of the Alter-Ego platform in routine clinical practice (see Supplementary Material S2 *)*. Figure 2 workflow describes the transformation of stakeholder suggestions into candidate robotic use cases. The descriptive quantitative data documenting the number of proposed tasks and their stakeholder of origin are reported in the matrix columns in the Supplementary Material S1. The number of suggestions should not be interpreted as a measure of the relative importance of each stakeholder group or their numeric representation in the focus sessions, but rather as a descriptive representation of the ideas generated within each professional perspective.

The qualitative analysis of stakeholder suggestions identified six functional clusters (Column 1): 1) Telepresence, Televisit, Teleassistance, Telerehabilitation; 2) User-related delivery services; 3) Guidance and welcome assistance; 4) Monitoring and security; 5) ADL (Activities of Daily Living) support and assistance tasks; and 6) Rehabilitation support. Consolidation of duplicate and conceptually similar suggestions resulted in 18 application proposals.

### 3.2 Feasibility analysis

For each proposed task, the associated technologies were identified and grouped into nine major categories (Figure 2, Column C).

These technologies, either individually or in combination, provide the basis for implementing the identified use cases. Their current maturity level and the time required to achieve the required technological development or system integration determined the feasibility of each task (Figure 2, Column C). Teleoperation had already been successfully tested with the Alter-Ego robot. Autonomous navigation, speech recognition, and tour operation have been implemented before the pilot study. Autonomous manipulation is currently under development, whereas keyword detection, transcription, image recognition, and text recognition require further implementation before being integrated into Alter-Ego.

Four proposed tasks (eye-tracking control, patient feeding, home ADL assistance, and patient mobilization) were excluded after technological feasibility assessment because the required technological developments could not be completed within the project timeframe.

During the subsequent multidisciplinary discussion, two technically feasible proposals (autonomous management of patient calls and automatic recording of clinical notes) were excluded because of ethical, organizational, regulatory, privacy, or intended-use considerations. Patient monitoring was retained as an exploratory outcome. (see Discussion).

This process resulted in the selection of 12 candidate use cases for experimental protocol implementation.

For each selected task, the relevant stakeholder groups were designated to be enrolled in the future trial (Supplementary Material).

### 3.3 Exploratory pilot phase

Representative photographs of the activities performed during the experimental sessions are presented in Figure 3, whereas Figure 4 summarizes the results of the exploratory pilot phase. Overall, both healthcare professionals and patients self-evaluated a positive experience when interacting with the Alter-Ego platform.

**FIGURE 3:**
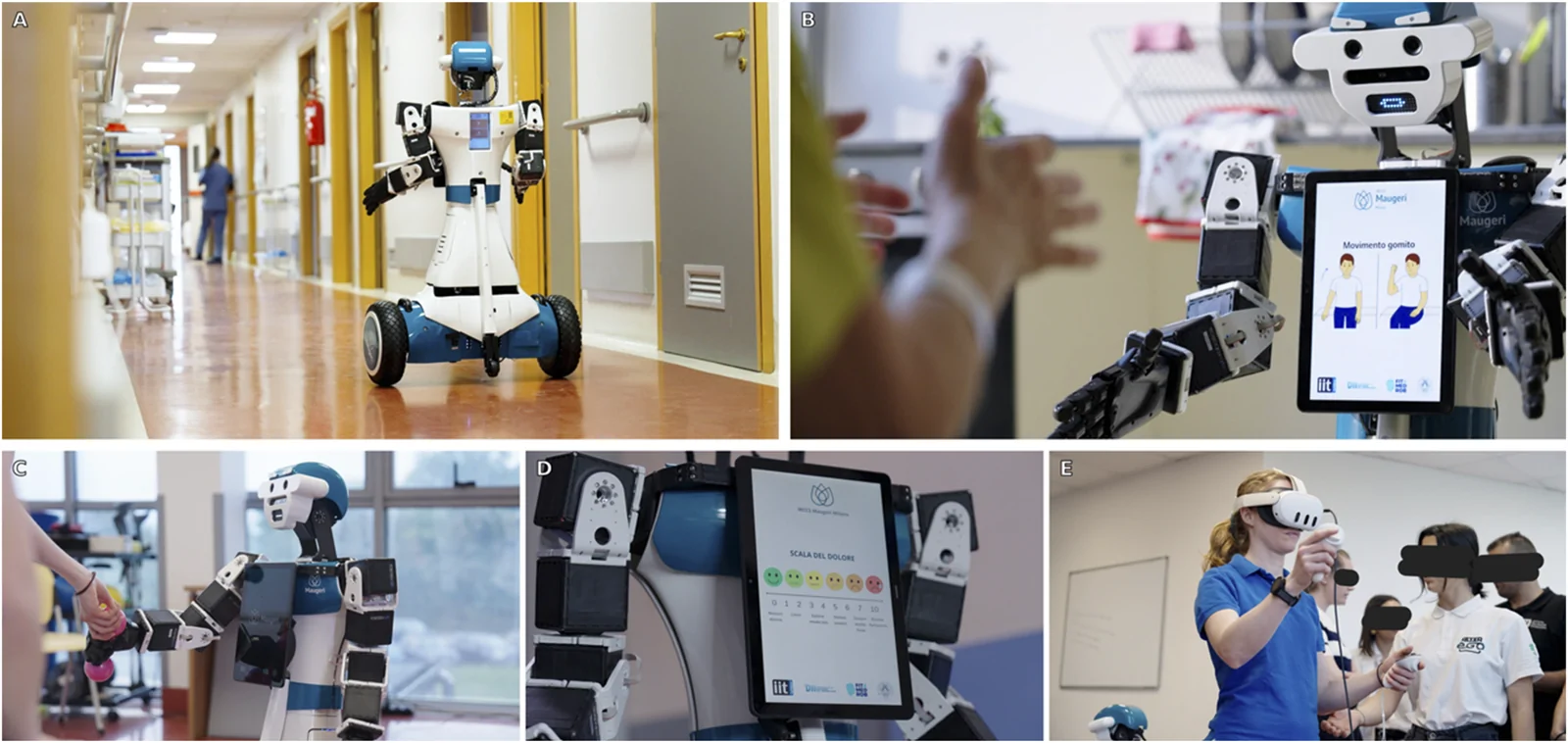
Alter-Ego platform and representative experimental activities. **(A)** Alter-Ego operating in the hospital environment. **(B)** Visual support for physiotherapy exercises through the robot-mounted display. **(C)** Object manipulation and delivery enabled by the robot’s upper limbs and hands. **(D)** Visual Analogue Scale displayed for patient-reported symptom assessment. **(E)** Operator interface used to teleoperate Alter-Ego through a head-mounted display and handheld controllers.

**FIGURE 4:**
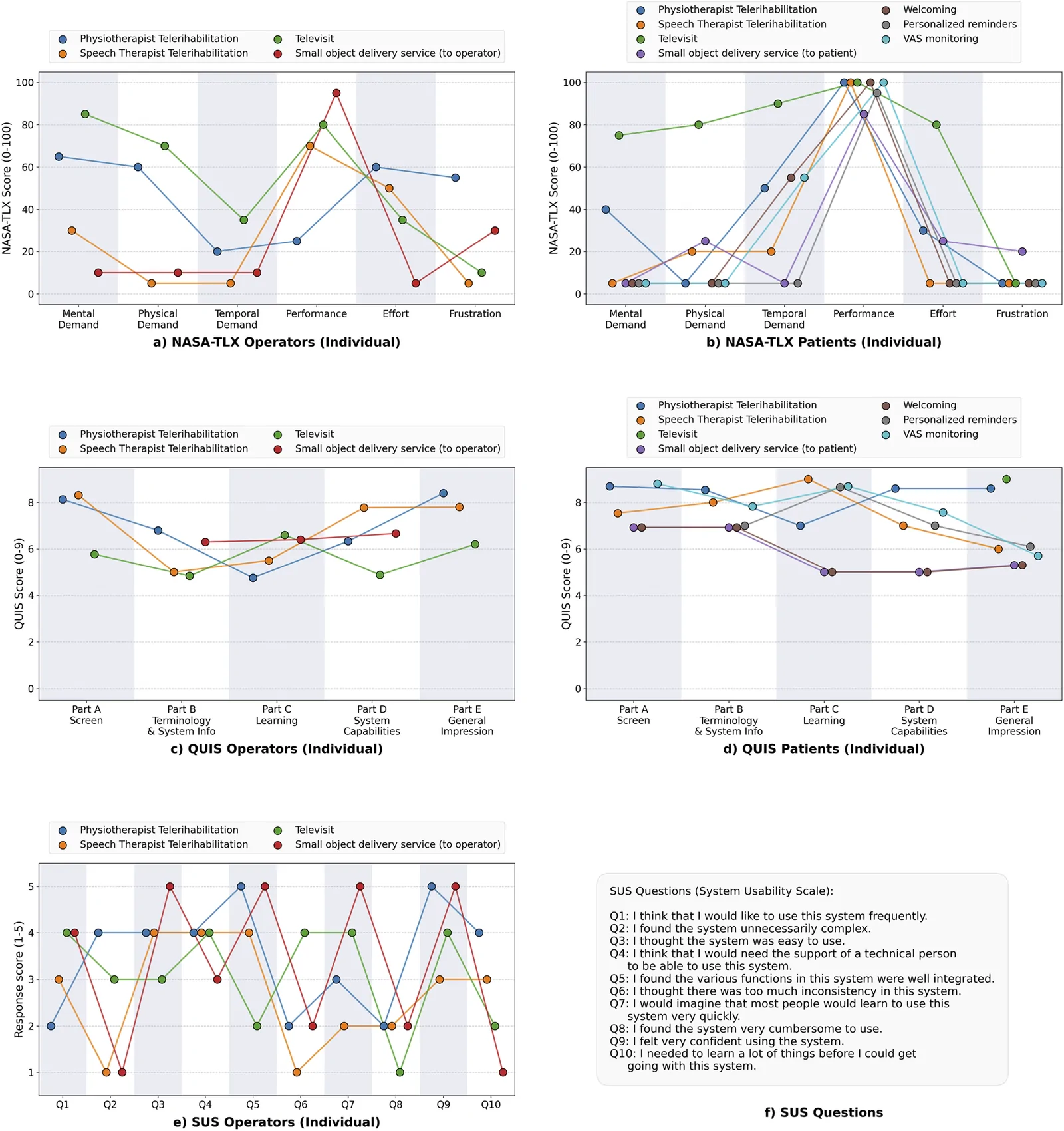
Results of the exploratory pilot phase. The panels present the exploratory feedback collected from healthcare professionals and patients using standardized questionnaires. **(a)** Individual raw scores of the NASA Task Load Index (NASA-TLX) reported by healthcare professionals, and **(b)** by patients, indicating perceived workload across different dimensions. Scores were converted to a 0–100 scale. The Performance dimension was recoded so that higher scores consistently indicate better perceived performance, whereas higher scores in the other dimensions indicate greater perceived workload. **(c)** Individual Questionnaire for User Interface Satisfaction (QUIS) dimension scores reported by healthcare professionals and **(d)** by patients across the evaluated system dimensions. QUIS scores range from 1 to 9, with higher values indicating greater satisfaction. Not all QUIS dimensions are reported for every task because the questionnaire included a “not applicable” response option for items that were not relevant to the participant’s role, the specific use case, or the interface with which the participant interacted. **(e)** Item-level responses to the System Usability Scale (SUS) completed by healthcare professionals. Responses range from 1 to 5 and are presented without calculation of an overall SUS score to identify task-specific usability strengths and critical issues. **(f)** Wording of the ten SUS items. From Q1 to Q10. Colours identify the specific use case evaluated by each participant.

Figures 4a,b report the individual NASA-TLX subscale scores for healthcare professionals and patients, respectively. Subjective workload was assessed using the Raw NASA-TLX (RTLX) (Georgsson, 2020). We analyzed raw, unweighted scores across the six dimensions using a 20-point scale. The anchors for Mental Demand, Physical Demand, Temporal Demand, Effort, and Frustration ranged from ‘Very Low’ to ‘Very High’. To facilitate intuitive interpretation, the Performance dimension was coded such that higher scores consistently indicate better performance. This directionality is maintained throughout our analysis and figures. Analyzing the individual components rather than an aggregate average reveals distinct workload profiles across the different use cases. For the healthcare professionals (Figure 4a), the Televisit and Physiotherapist Telerehabilitation tasks exhibited the highest Mental (approx. 85 and 65) and Physical Demands (approx. 70 and 60). Notably, the Physiotherapist task also registered the highest levels of Effort and Frustration among all operator activities. In contrast, the *Small object delivery* and *Speech Therapist Telerehabilitation* tasks demanded notably less physical and mental exertion. For the patients (Figure 4b), the workload was generally low across most subscales for the majority of the tasks. However, *Televisit* emerged as a significant outlier, demanding substantially higher Mental, Physical, and Temporal Demands, as well as higher Effort compared to simpler tasks like *Welcoming* or *VAS monitoring*. Interestingly, despite these varying demand levels, the self-evaluated performance score was consistently high (between 80 and 100) across nearly all tasks for patients, indicating a strong perceived success in task execution.

The QUIS results (Figures 4c,d) demonstrated a preliminary positive perception of the interaction with the platform. Specifically, the evaluated interfaces varied depending on the stakeholder and the task. During teleoperation tasks, healthcare professionals engaged with a dedicated teleoperation interface. Conversely, patients consistently interacted directly with the physical robot and a tablet mounted on its torso. Ratings were high across all evaluated dimensions, including overall reaction to the system (Dimension E), screen/interface characteristics (Dimension A), terminology and system information (Dimension B), learning (Dimension C), and system capabilities (Dimension D). Most scores ranged between 5 and 8.5 among healthcare professionals and between 6 and 9 among patients.

The item-level analysis revealed task-specific differences that would not have been captured by an overall usability score. Among healthcare professionals (Figure 4c), slightly lower ratings were observed for screen/interface characteristics during the speech therapy use case, for terminology and system information during both telerehabilitation use cases, for learning during the televisit task, and for system capabilities during the speech therapy task. Among patients (Figure 4d), lower scores were reported for the learning, system capabilities, and overall reaction dimensions during the delivery services and personalized reminder use cases, while a slight reduction in the overall reaction score was also observed during the VAS monitoring task. Nevertheless, all ratings remained within the positive range, and no major usability concerns were reported in this preliminary evaluation.

The SUS is presented in Figure 4e, while the ten questions of the questionnaire are presented in panel f of the same figure. Please note that the SUS scores presented in this analysis are raw values. Consequently, higher scores on odd-numbered items indicate positive feedback, whereas higher scores on even-numbered items indicate negative feedback.

SUS item-level analysis similarly highlighted differences among individual use cases rather than generalized limitations of the platform. Feedback indicated a generally positive trend across the evaluated tasks; however, greater variability emerged in items exploring system complexity (Q2), consistency (Q6), and the amount of learning required before using the platform (Q10).

## 4 Discussion

### 4.1 Focus group and feasibility analysis

The present study illustrates how the potential integration of a versatile robotic platform can be explored within a multidisciplinary neurological rehabilitation setting. It highlights the value of a multidisciplinary and inclusive approach involving engineers, healthcare providers, patients, and caregivers to ensure that the development and deployment of robotic solutions are aligned with the complex and heterogeneous needs of real-world clinical practice. 81 stakeholder suggestions were identified, demonstrating the platform’s versatility and relevance across different healthcare and domestic environments.

Although stakeholder groups were not numerically balanced, the multidisciplinary clinical team was almost fully represented, including all physicians, speech therapists, and occupational therapists, and most physiotherapists involved in ALS care. The smaller number of patients and caregivers may limit the breadth and generalizability of their perspectives. However, the study primarily aimed to identify healthcare professionals’ needs while incorporating patient and caregiver views, as key stakeholders, to assess the alignment between clinical requirements and care expectations. The number and diversity of use cases proposed suggest that stakeholders were able to envision the platform within their working or care environment. However, the participatory process focused on identifying and prioritizing applications rather than systematically assessing attitudes, implementation barriers, or determinants of acceptance. Alter-Ego’s anthropomorphic design may have contributed to the favourable initial reactions, although the study was not designed to assess the specific influence of robot morphology. These aspects will be further investigated in the subsequent clinical trial.

The identified functional clusters address key challenges in healthcare delivery and outline how the Alter-Ego platform might support selected clinical activities, particularly routine and time-consuming tasks. Such support could help healthcare professionals to devote more time to patients and caregivers in activities requiring human expertise, judgement, and empathy - qualities that remain inherently human and cannot be replaced by robotic systems.

As this work was intentionally designed as a single-centre case study, the identified use cases and their prioritization may reflect the organizational characteristics and clinical workflows of our ALS unit. Future studies across different healthcare settings will be important to evaluate the transferability of the proposed methodological framework and the feasibility and actual organizational impact of the proposed applications.

The feasibility analysis represented a key component of this methodology, critically examining the technological readiness, safety implications, and clinical, ethical, and regulatory considerations of each proposed task. In the investigated context, this process helped characterize the alignment between stakeholder needs and the current capabilities and limitations of the Alter-Ego platform.

In particular, the joint feasibility analysis highlighted several additional considerations beyond technical feasibility. Although some applications could be envisaged in future developments, those involving direct physical interaction with patients were considered unsuitable because of clinical risks, professional responsibilities, and insurance implications. For example, autonomous feeding could expose patients with dysphagia to aspiration or choking, whereas robotic mobilization could cause injury. These activities also lacked consensus among healthcare professionals, who considered direct patient handling an essential component of care. Other proposals were excluded for organizational or regulatory constraints. Autonomous management of patient calls required a level of decision-making that was not considered sufficiently safe. Automatic recording of clinical notes, although technically feasible, was not prioritized because of the development effort, limited perceived benefit, and unresolved integration, privacy, and data-management issues. Autonomous monitoring of physiological parameters was also excluded because it would have changed Alter-Ego’s intended use from an assistive platform to a medical device. However, fall-risk monitoring was retained as an exploratory outcome because of its high clinical relevance, although it requires further development and validation.

The ethical debate surrounding humanoid and assistive robots in healthcare extends beyond safety and technical performance. Recurrent concerns include the risks of reduced human contact, loss of privacy and autonomy, and deceptive or infantilizing interactions, as well as the broader questions of dignity, responsibility, and the fair distribution of benefits and burdens (Sharkey and Sharkey, 2012; Elendu et al., 2023). Elsewhere, adopting an ethics-of-care perspective, robots should strengthen rather than undermine relationships among patients, caregivers, and healthcare professionals (Yuan et al., 2023). Accordingly, trust, privacy, safety, staff training, organizational readiness, accountability, and data protection are key considerations for the responsible implementation of humanoid robots in hospital and social-care settings (Papadopoulos et al., 2020). Finally, the feasibility analysis reflects the current technological landscape. The dynamic advancements in robotics and artificial intelligence suggest that tasks considered infeasible today may become viable, making continuous stakeholder involvement essential to guide clinically relevant development.

### 4.2 Exploratory pilot phase

The exploratory feedback collected during this pilot phase provided preliminary but valuable insights into the current maturity of the Alter-Ego platform and the feasibility of the stakeholder-driven use cases. Given the exploratory nature of the study and the limited sample, the findings should not be interpreted as evidence of efficacy or platform limitations, but as indications for refining the robotic system and the subsequent clinical trial protocol. NASA-TLX findings indicate an acceptable overall workload across participants, an expected and positive outcome given their brief initial familiarization with the platform. However, breaking down the scores by individual tasks provides deeper insights into the usability dynamics. For the healthcare professionals, the prominent peaks in Mental and Physical Demands observed during the teleoperated tasks likely reflected the head-mounted teleoperation interface, which required simultaneous robot control, audiovisual communication, and clinical observation. This multi-level cognitive and physical engagement is naturally more demanding than conventional computer-based interfaces. Importantly, these task-specific demands were not associated with increased Frustration levels, suggesting that the additional workload was perceived as manageable. Patients’ workload was generally even lower, with *Televisit* being the only outlier requiring higher cognitive and physical engagement compared to routine assistance tasks such as small-object delivery. Despite these differences, patients assigned the highest possible scores to the Performance dimension across nearly all activities with no major usability concerns in the tested conditions.

QUIS findings confirmed generally positive initial perceptions while identifying task-specific areas for improvement.

During the speech-therapy scenario, lower ratings for interface characteristics and system capabilities were consistent with therapists’ reports that the current audiovisual quality was insufficient for detailed assessment of speech and swallowing, where subtle clinical signs are essential. Nevertheless, the platform was considered potentially suitable for follow-up, monitoring, and rehabilitation activities, requiring less detailed audiovisual information. Lower scores for terminology and system information during the telerehabilitation also suggest the need for more intuitive commands and clearer system feedback. Lower learning scores should also be interpreted in relation to the safety procedures adopted during this preliminary phase. Healthcare professionals operated the robot only under engineering supervision, while initialization, configuration, and safety procedures remained the responsibility of technical staff. However, independent robot use was not the primary objective of this exploratory phase, which was specifically designed to validate the selected use cases, refine the study protocol, and identify early issues. Accordingly, the evaluation protocol included specific questionnaire items investigating participants’ perceived ability to operate the platform independently and the level of technical support required. The findings therefore reflect the current need for engineering support rather than the healthcare professionals’ ability to perform the selected clinical tasks. They nevertheless highlight the importance of simplifying deployment procedures and progressively increasing operator autonomy before subsequent clinical trials.

Lower overall reaction scores during VAS monitoring may instead reflect the limited immediate benefit perceived by patients from repeated pain assessment, despite its clinical relevance. This discrepancy among patients - lower ratings for learning, system capabilities, and overall reaction - was mainly associated with delivery services and personalized reminders. Unlike telepresence applications, these tasks required autonomous navigation and physically delivering objects or information, which may have generated uncertainty regarding the robot’s behaviour or capabilities during the first exposure, leading to slightly lower satisfaction despite the absence of major usability concerns.

Lower overall reaction scores during the VAS monitoring may instead reflect the limited immediate benefit perceived by patients from repeated pain monitoring despite its clinical relevance - it represents a mandatory component of routine hospital care. This discrepancy illustrates how patient-perceived usefulness may differ from professional and organizational priorities, supporting the involvement of multiple stakeholder groups in the design process.

Overall, the variations observed were linked to specific services rather than to generalized dissatisfaction with the platform. This is particularly relevant for a versatile system such as Alter-Ego, whose value lies in the ability to support multiple clinical activities through a single platform. Unlike conventional studies evaluating a robot designed for a single task, the present approach makes it possible to compare heterogeneous use cases and identify targeted improvements of individual functionalities instead of generic modifications to the platform.

Finally, perceptions were collected during participants’ first interaction with Alter-Ego in a controlled research setting and may have been influenced by technological novelty; some degree of social-desirability effect cannot be excluded. They may therefore evolve with greater familiarity and prolonged clinical use. Overall, these preliminary findings support the stakeholder-driven methodology as a useful approach for identifying feasible applications, detecting task-specific usability issues, and guiding the refinement of both the platform and the clinical trial protocol.

## 5 Conclusions

To our knowledge, this study provides an early example of the development of a clinical trial involving a versatile, multi-purpose humanoid robot designed to support multiple healthcare services rather than a single predefined task. The main contribution is the description of a structured, stakeholder-driven process for matching clinical needs with the technological capabilities and identifying meaningful and technically feasible applications for rehabilitation and assistance. By integrating stakeholders’ perspectives with clinical needs, technical and infrastructural considerations, the proposed methodology supported use-case prioritization and protocol development. The subsequent exploratory evaluation provided initial descriptive feedback on the acceptability and identified task-specific areas requiring technological and procedural refinement of the experimental protocol before larger clinical studies.

This bidirectional process encourages healthcare professionals to critically reflect on their clinical needs, workflows, priorities, and future roles in increasingly technology-rich environments, while providing technology developers with direct insight into real-world clinical challenges. By focusing the development on applications considered valuable by future users, it may improve usability, acceptance, workflow integration, and the likelihood of successful real-world implementation.

Although developed within a multidisciplinary ALS care setting, the proposed process may inform similar initiatives involving emerging robotic technologies. Its application should be adapted according to the clinical population, organizational context, available infrastructure, and characteristics of the robotic platform. For example, several requirements and applications identified in this study may also be relevant to elderly care, although their transfer to older and frail populations would require specific consideration of cognitive and sensory impairment, multimorbidity, home environments, and caregiver involvement.The future of healthcare robotics will depend not only on building smarter robots, but also on designing smarter pathways for their clinical evaluation, acceptance, and integration into everyday care.

## Data Availability

The raw data supporting the conclusions of this article will be made available by the authors upon request.

## Footnotes

1
https://www.naturalmachinemotioninitiative.com/
