# Supplementary material for: Bridging stakeholder needs and technological capabilities to identify humanoid robot applications in rehabilitation: a case study in an ALS multidisciplinary care unit
Source: Front Robot AI. 2026 Sep 21;13:1944029. doi: 10.3389/frobt.2026.1944029 (PMC13634871; doi:10.3389/frobt.2026.1944029)
Supplement: Supplementary file 1 [file Supplementaryfile2.docx]

**Supplementary Table S1. Stakeholder-generated suggestions**

*Standardised reconstruction of 81 stakeholder contributions from contemporaneous meeting reports and the study coding matrix*

**Methodological note.** The entries below preserve the content, stakeholder attribution and frequency recorded in the study report and coding matrix. They are not verbatim transcript quotations: wording was translated into English and minimally standardised for clarity, and overlapping contributions remain listed separately. The distribution is: medical doctors (n=16), physiotherapists (n=23), healthcare assistants (n=8), patients (n=15), caregivers (n=2), speech therapists (n=3), and nurses (n=14).

| **ID** | **Stakeholder group** | **Standardised stakeholder suggestion** | **Corresponding consolidated application** | **Functional category** |
| --- | --- | --- | --- | --- |
| S01 | Medical doctors | Use ALTER-EGO to conduct remote medical consultations with patients in hospital or at home. | Teleconsultations or televisits | Telepresence, televisit, teleassistance and telerehabilitation |
| S02 | Medical doctors | Enable the remote physician to observe the patient from different positions and angles, including at the bedside. | Teleconsultations or televisits | Telepresence, televisit, teleassistance and telerehabilitation |
| S03 | Medical doctors | Use ALTER-EGO for specialist teleconsultation while another professional remains physically present with the patient. | Teleconsultations or televisits | Telepresence, televisit, teleassistance and telerehabilitation |
| S04 | Medical doctors | Allow the remote physician to follow the patient during movement and use the robot’s gestures or pointing capabilities during assessment. | Teleconsultations or televisits | Telepresence, televisit, teleassistance and telerehabilitation |
| S05 | Physiotherapists | Use ALTER-EGO for remote physiotherapy consultations and functional observation. | Teleconsultations or televisits | Telepresence, televisit, teleassistance and telerehabilitation |
| S06 | Physiotherapists | Allow a remote therapist to follow and observe the patient while moving through the environment. | Teleconsultations or televisits | Telepresence, televisit, teleassistance and telerehabilitation |
| S07 | Physiotherapists | Use the robot’s embodied presence to demonstrate, gesture or point during remote rehabilitation encounters. | Teleconsultations or televisits | Telepresence, televisit, teleassistance and telerehabilitation |
| S08 | Patients | Use ALTER-EGO to access medical teleconsultations from home. | Teleconsultations or televisits | Telepresence, televisit, teleassistance and telerehabilitation |
| S09 | Speech therapists | Use ALTER-EGO to support remote assessment of swallowing. | Teleconsultations or televisits | Telepresence, televisit, teleassistance and telerehabilitation |
| S10 | Speech therapists | Use ALTER-EGO for remote speech and language therapy assessment. | Teleconsultations or televisits | Telepresence, televisit, teleassistance and telerehabilitation |
| S11 | Medical doctors | Provide remote support during the initial transition home and verify adherence to prescribed procedures. | Remote assistance in the use of medical devices or procedures | Telepresence, televisit, teleassistance and telerehabilitation |
| S12 | Medical doctors | Guide patients or caregivers remotely in the use of respiratory support equipment. | Remote assistance in the use of medical devices or procedures | Telepresence, televisit, teleassistance and telerehabilitation |
| S13 | Medical doctors | Guide patients or caregivers remotely in procedures related to PEG management. | Remote assistance in the use of medical devices or procedures | Telepresence, televisit, teleassistance and telerehabilitation |
| S14 | Physiotherapists | Provide remote assistance with the setup and use of rehabilitation or respiratory devices at home. | Remote assistance in the use of medical devices or procedures | Telepresence, televisit, teleassistance and telerehabilitation |
| S15 | Patients | Receive remote guidance when learning to use medical or assistive equipment at home. | Remote assistance in the use of medical devices or procedures | Telepresence, televisit, teleassistance and telerehabilitation |
| S16 | Medical doctors | Use ALTER-EGO to remotely inspect the home environment when planning assistive devices and adaptations. | Home assessment for assistive devices and space reorganisation | Telepresence, televisit, teleassistance and telerehabilitation |
| S17 | Physiotherapists | Map the patient’s home so therapists can remotely identify barriers, required aids and possible space reorganisation. | Home assessment for assistive devices and space reorganisation | Telepresence, televisit, teleassistance and telerehabilitation |
| S18 | Physiotherapists | Deliver and supervise physiotherapy exercises remotely in the patient’s home. | Home support for telerehabilitation | Telepresence, televisit, teleassistance and telerehabilitation |
| S19 | Patients | Use ALTER-EGO at home to perform maintenance exercises with remote professional support. | Home support for telerehabilitation | Telepresence, televisit, teleassistance and telerehabilitation |
| S20 | Speech therapists | Deliver remote speech and language therapy sessions through ALTER-EGO. | Home support for telerehabilitation | Telepresence, televisit, teleassistance and telerehabilitation |
| S21 | Medical doctors | Retrieve and deliver a patient’s clinical record when requested by a physician. | Transport of small items for healthcare professionals | User-related delivery services |
| S22 | Physiotherapists | Bring balls or other small rehabilitation objects to the therapist or patient. | Transport of small items for healthcare professionals | User-related delivery services |
| S23 | Physiotherapists | Bring small weights required during rehabilitation exercises. | Transport of small items for healthcare professionals | User-related delivery services |
| S24 | Physiotherapists | Deliver instruments or accessories needed for a rehabilitation session. | Transport of small items for healthcare professionals | User-related delivery services |
| S25 | Physiotherapists | Transport small monitoring devices between the ward and rehabilitation areas. | Transport of small items for healthcare professionals | User-related delivery services |
| S26 | Physiotherapists | Bring respiratory or rehabilitation equipment to the patient’s room when required. | Transport of small items for healthcare professionals | User-related delivery services |
| S27 | Nurses | Transport small ward items, such as documents or test tubes, on request. | Transport of small items for healthcare professionals | User-related delivery services |
| S28 | Healthcare assistants | Retrieve food or water requested by a patient. | Delivery of small personal items to patients | User-related delivery services |
| S29 | Healthcare assistants | Pick up and return small personal items that have fallen to the floor. | Delivery of small personal items to patients | User-related delivery services |
| S30 | Healthcare assistants | Retrieve frequently used bedside objects, such as a remote control or telephone. | Delivery of small personal items to patients | User-related delivery services |
| S31 | Patients | Pick up dropped objects such as cutlery, clothing, tissues, books or electronic devices. | Delivery of small personal items to patients | User-related delivery services |
| S32 | Patients | Pass objects located on or inside the bedside table, such as a telephone, water or a remote control. | Delivery of small personal items to patients | User-related delivery services |
| S33 | Patients | Bring clothing and linen to the bathroom. | Delivery of small personal items to patients | User-related delivery services |
| S34 | Patients | Fetch a drink or coffee from another area. | Delivery of small personal items to patients | User-related delivery services |
| S35 | Caregivers | Retrieve and deliver small household or personal objects to reduce the caregiver’s practical workload. | Delivery of small personal items to patients | User-related delivery services |
| S36 | Nurses | Bring food or water to a patient when ward staff are occupied. | Delivery of small personal items to patients | User-related delivery services |
| S37 | Nurses | Collect a small requested item from another hospital area, such as a snack from the bar. | Delivery of small personal items to patients | User-related delivery services |
| S38 | Medical doctors | Record voice notes dictated by physicians during ward rounds. | Voice-note recording and transcription during clinical rounds | User-related delivery services |
| S39 | Medical doctors | Transcribe dictated clinical observations into an electronic file for subsequent documentation. | Voice-note recording and transcription during clinical rounds | User-related delivery services |
| S40 | Nurses | Record and transcribe nursing notes during ward activities. | Voice-note recording and transcription during clinical rounds | User-related delivery services |
| S41 | Medical doctors | Guide patients who have difficulty orienting themselves within the hospital. | Guidance and accompaniment | Guidance and welcome assistance |
| S42 | Physiotherapists | Accompany patients from their hospital room to the rehabilitation gym. | Guidance and accompaniment | Guidance and welcome assistance |
| S43 | Physiotherapists | Guide patients back to their room after rehabilitation. | Guidance and accompaniment | Guidance and welcome assistance |
| S44 | Physiotherapists | Accompany patients with mobility difficulties along scheduled hospital routes. | Guidance and accompaniment | Guidance and welcome assistance |
| S45 | Patients | Accompany the patient through the hospital and assist with route finding or doors along the way. | Guidance and accompaniment | Guidance and welcome assistance |
| S46 | Nurses | Accompany patients between the activities scheduled during a complex outpatient care day. | Guidance and accompaniment | Guidance and welcome assistance |
| S47 | Medical doctors | Welcome patients and provide an initial orientation to the clinical service. | Reception and welcome procedures | Guidance and welcome assistance |
| S48 | Medical doctors | Present standard admission information or the service charter to newly arrived patients. | Reception and welcome procedures | Guidance and welcome assistance |
| S49 | Patients | Explain hospital procedures and provide additional information that the patient does not understand. | Reception and welcome procedures | Guidance and welcome assistance |
| S50 | Nurses | Welcome patients attending the day-service pathway and explain the planned sequence of appointments. | Reception and welcome procedures | Guidance and welcome assistance |
| S51 | Nurses | Update and communicate changes in the order or timing of scheduled examinations and rehabilitation sessions. | Reception and welcome procedures | Guidance and welcome assistance |
| S52 | Medical doctors | Provide personalised reminders related to prescribed activities or treatments. | Personalised reminders for daily activities | Guidance and welcome assistance |
| S53 | Physiotherapists | Remind patients of scheduled rehabilitation sessions. | Personalised reminders for daily activities | Guidance and welcome assistance |
| S54 | Physiotherapists | Prompt patients when it is time to begin a planned exercise programme. | Personalised reminders for daily activities | Guidance and welcome assistance |
| S55 | Physiotherapists | Provide timing reminders during the patient’s daily rehabilitation schedule. | Personalised reminders for daily activities | Guidance and welcome assistance |
| S56 | Patients | Remind the patient to take prescribed medication. | Personalised reminders for daily activities | Guidance and welcome assistance |
| S57 | Nurses | Remind patients of meals and other routine ward activities. | Personalised reminders for daily activities | Guidance and welcome assistance |
| S58 | Nurses | Provide personalised reminders according to each patient’s daily clinical agenda. | Personalised reminders for daily activities | Guidance and welcome assistance |
| S59 | Healthcare assistants | Use ALTER-EGO as a mobile camera to check whether a patient remains safely in bed. | Patient monitoring and fall detection | Monitoring and security |
| S60 | Healthcare assistants | Detect a possible fall during routine ward monitoring. | Patient monitoring and fall detection | Monitoring and security |
| S61 | Healthcare assistants | Read or relay values displayed on the patient’s clinical monitor. | Patient monitoring and fall detection | Monitoring and security |
| S62 | Healthcare assistants | Perform scheduled daytime or night-time checks of patients in their rooms. | Patient monitoring and fall detection | Monitoring and security |
| S63 | Nurses | Conduct scheduled night rounds to check breathing, monitored parameters and possible falls. | Patient monitoring and fall detection | Monitoring and security |
| S64 | Medical doctors | Administer clinical questionnaires and rating scales through the robot interface. | Administration of questionnaires and rating scales | Monitoring and security |
| S65 | Physiotherapists | Collect patient-reported ratings relevant to rehabilitation sessions. | Administration of questionnaires and rating scales | Monitoring and security |
| S66 | Physiotherapists | Administer the pain visual analogue scale before or after rehabilitation activities. | Administration of questionnaires and rating scales | Monitoring and security |
| S67 | Nurses | Collect the pain VAS through the robot-mounted interface. | Administration of questionnaires and rating scales | Monitoring and security |
| S68 | Nurses | Repeat a patient-reported assessment when a previously recorded value exceeds a predefined threshold. | Administration of questionnaires and rating scales | Monitoring and security |
| S69 | Healthcare assistants | Use ALTER-EGO as an intercom to make initial contact after a patient activates the call bell. | Response to patient calls and call allocation | Monitoring and security |
| S70 | Nurses | Assess the urgency of a patient call through audio-video communication and available monitor information. | Response to patient calls and call allocation | Monitoring and security |
| S71 | Nurses | Route the patient’s request to the appropriate nurse or healthcare assistant. | Response to patient calls and call allocation | Monitoring and security |
| S72 | Physiotherapists | Assist with feeding a patient who is unable to bring food independently to the mouth. | Patient feeding | ADL support and assistance |
| S73 | Patients | Assist with opening doors and moving objects that obstruct movement at home. | Home assistance with activities of daily living | ADL support and assistance |
| S74 | Patients | Support simple domestic activities such as tidying, retrieving clothing or preparing for washing. | Home assistance with activities of daily living | ADL support and assistance |
| S75 | Patients | Allow a patient with severe motor impairment to control ALTER-EGO using an eye-tracking system. | Patient control through an eye-tracking interface | ADL support and assistance |
| S76 | Patients | Adjust wheelchair posture, including backrest tilt and leg position, when requested by the patient. | Postural adjustment and limb mobilisation | ADL support and assistance |
| S77 | Patients | Mobilise an upper limb or help the patient scratch or remove an insect. | Postural adjustment and limb mobilisation | ADL support and assistance |
| S78 | Caregivers | Assist with demanding mobility tasks, such as transfers between the bed and wheelchair. | Postural adjustment and limb mobilisation | ADL support and assistance |
| S79 | Physiotherapists | Count repetitions and provide timing cues during individual rehabilitation exercises. | Support during rehabilitation exercises | Rehabilitation support |
| S80 | Physiotherapists | Display or demonstrate exercises during group rehabilitation while the therapist assists patients requiring closer supervision. | Support during rehabilitation exercises | Rehabilitation support |
| S81 | Physiotherapists | Support walking or respiratory rehabilitation by observing task execution and recording values communicated during the exercise. | Support during rehabilitation exercises | Rehabilitation support |
